# Supplementary material for: Developing and validating the Japanese version of the Referential Thinking Scale: A cross-sectional study
Source: PLoS One. 2023 Jul 7;18(7):e0283416. doi: 10.1371/journal.pone.0283416 (PMC10328373; doi:10.1371/journal.pone.0283416)
Supplement: S1 Table — (DOCX) [file pone.0283416.s001.docx]

**S1 Table. Descriptive statistics of the J-REF (n = 600)**

| **Item Number** | **n** | **Means** | **SD** | **Min.** | **Max.** | **Skewness** | **Kurtosis** |
| --- | --- | --- | --- | --- | --- | --- | --- |
| **1** | 600 | 0.39 | 0.49 | 0 | 1 | 0.46 | -1.79 |
| **2** | 600 | 0.16 | 0.36 | 0 | 1 | 1.88 | 1.55 |
| **3** | 600 | 0.27 | 0.44 | 0 | 1 | 1.05 | -0.89 |
| **4** | 600 | 0.07 | 0.26 | 0 | 1 | 3.22 | 8.38 |
| **5** | 600 | 0.06 | 0.23 | 0 | 1 | 3.83 | 12.65 |
| **6** | 600 | 0.11 | 0.31 | 0 | 1 | 2.54 | 4.47 |
| **7** | 600 | 0.05 | 0.21 | 0 | 1 | 4.20 | 15.68 |
| **8** | 600 | 0.13 | 0.33 | 0 | 1 | 2.24 | 3.02 |
| **9** | 600 | 0.32 | 0.47 | 0 | 1 | 0.76 | -1.42 |
| **10** | 600 | 0.06 | 0.23 | 0 | 1 | 3.83 | 12.65 |
| **11** | 600 | 0.09 | 0.29 | 0 | 1 | 2.82 | 5.98 |
| **12** | 600 | 0.06 | 0.23 | 0 | 1 | 3.89 | 13.19 |
| **13** | 600 | 0.01 | 0.11 | 0 | 1 | 9.07 | 80.45 |
| **14** | 600 | 0.17 | 0.38 | 0 | 1 | 1.72 | 0.97 |
| **15** | 600 | 0.01 | 0.10 | 0 | 1 | 9.82 | 94.68 |
| **16** | 600 | 0.18 | 0.38 | 0 | 1 | 1.69 | 0.86 |
| **17** | 600 | 0.07 | 0.26 | 0 | 1 | 3.36 | 9.32 |
| **18** | 600 | 0.10 | 0.30 | 0 | 1 | 2.60 | 4.77 |
| **19** | 600 | 0.33 | 0.47 | 0 | 1 | 0.71 | -1.50 |
| **20** | 600 | 0.02 | 0.13 | 0 | 1 | 7.53 | 54.82 |
| **21** | 600 | 0.06 | 0.24 | 0 | 1 | 3.58 | 10.81 |
| **22** | 600 | 0.04 | 0.19 | 0 | 1 | 4.92 | 22.23 |
| **23** | 600 | 0.02 | 0.15 | 0 | 1 | 6.55 | 41.03 |
| **24** | 600 | 0.07 | 0.26 | 0 | 1 | 3.36 | 9.32 |
| **25** | 600 | 0.04 | 0.21 | 0 | 1 | 4.38 | 17.20 |
| **26** | 600 | 0.06 | 0.25 | 0 | 1 | 3.52 | 10.41 |
| **27** | 600 | 0.06 | 0.23 | 0 | 1 | 3.89 | 13.19 |
| **28** | 600 | 0.14 | 0.35 | 0 | 1 | 2.05 | 2.21 |
| **29** | 600 | 0.20 | 0.40 | 0 | 1 | 1.47 | 0.16 |
| **30** | 600 | 0.18 | 0.39 | 0 | 1 | 1.63 | 0.67 |
| **31** | 600 | 0.21 | 0.41 | 0 | 1 | 1.42 | 0.02 |
| **32** | 600 | 0.10 | 0.30 | 0 | 1 | 2.72 | 5.42 |
| **33** | 600 | 0.16 | 0.37 | 0 | 1 | 1.87 | 1.49 |
| **34** | 600 | 0.06 | 0.25 | 0 | 1 | 3.52 | 10.41 |

Note: N = 600. The scores of Item 19 (reverse item) was reversed.
