# Supplementary material for: Developing and validating the Japanese version of the Referential Thinking Scale: A cross-sectional study
Source: PLoS One. 2023 Jul 7;18(7):e0283416. doi: 10.1371/journal.pone.0283416 (PMC10328373; doi:10.1371/journal.pone.0283416)
Supplement: S2 Table — (DOCX) [file pone.0283416.s002.docx]

**S2 Table. Descriptive statistics of the J-REF and gender differences (n = 600).**

|  | **Male (n = 300)** | | | | | | |  | **Female (n = 300)** | | | | | | |  |
| --- | --- | --- | --- | --- | --- | --- | --- | --- | --- | --- | --- | --- | --- | --- | --- | --- |
| **Item number** | **n** | **Means** | **SD** | **Min.** | **Max.** | **Skewness** | **Kurtosis** |  | **n** | **Means** | **SD** | **Min.** | **Max.** | **Skewness** | **Kurtosis** | ***P* value** |
| **1** | 300 | 0.35 | 0.48 | 0 | 1 | 0.63 | -1.61 |  | 300 | 0.42 | 0.49 | 0 | 1 | 0.31 | -1.91 | n.s. |
| **2** | 300 | 0.14 | 0.35 | 0 | 1 | 2.06 | 2.27 |  | 300 | 0.17 | 0.38 | 0 | 1 | 1.72 | 0.95 | n.s. |
| **3** | 300 | 0.24 | 0.43 | 0 | 1 | 1.21 | -0.53 |  | 300 | 0.29 | 0.46 | 0 | 1 | 0.90 | -1.19 | n.s. |
| **4** | 300 | 0.09 | 0.28 | 0 | 1 | 2.92 | 6.57 |  | 300 | 0.06 | 0.24 | 0 | 1 | 3.57 | 10.76 | n.s. |
| **5** | 300 | 0.07 | 0.25 | 0 | 1 | 3.46 | 9.98 |  | 300 | 0.05 | 0.21 | 0 | 1 | 4.28 | 16.35 | n.s. |
| **6** | 300 | 0.07 | 0.26 | 0 | 1 | 3.26 | 8.64 |  | 300 | 0.14 | 0.35 | 0 | 1 | 2.06 | 2.27 | 0.00813 |
| **7** | 300 | 0.05 | 0.23 | 0 | 1 | 3.96 | 13.69 |  | 300 | 0.04 | 0.20 | 0 | 1 | 4.46 | 17.98 | n.s. |
| **8** | 300 | 0.11 | 0.32 | 0 | 1 | 2.43 | 3.91 |  | 300 | 0.14 | 0.35 | 0 | 1 | 2.06 | 2.27 | n.s. |
| **9** | 300 | 0.32 | 0.47 | 0 | 1 | 0.78 | -1.39 |  | 300 | 0.33 | 0.47 | 0 | 1 | 0.74 | -1.46 | n.s. |
| **10** | 300 | 0.05 | 0.23 | 0 | 1 | 3.96 | 13.69 |  | 300 | 0.06 | 0.24 | 0 | 1 | 3.69 | 11.63 | n.s. |
| **11** | 300 | 0.08 | 0.27 | 0 | 1 | 3.08 | 7.52 |  | 300 | 0.10 | 0.30 | 0 | 1 | 2.59 | 4.74 | n.s. |
| **12** | 300 | 0.06 | 0.24 | 0 | 1 | 3.69 | 11.63 |  | 300 | 0.05 | 0.22 | 0 | 1 | 4.11 | 14.93 | n.s. |
| **13** | 300 | 0.01 | 0.11 | 0 | 1 | 8.44 | 69.53 |  | 300 | 0.01 | 0.10 | 0 | 1 | 9.80 | 94.36 | n.s. |
| **14** | 300 | 0.17 | 0.38 | 0 | 1 | 1.75 | 1.06 |  | 300 | 0.18 | 0.38 | 0 | 1 | 1.69 | 0.85 | n.s. |
| **15** | 300 | 0.01 | 0.08 | 0 | 1 | 12.06 | 144.02 |  | 300 | 0.01 | 0.11 | 0 | 1 | 8.44 | 69.53 | n.s. |
| **16** | 300 | 0.18 | 0.38 | 0 | 1 | 1.69 | 0.85 |  | 300 | 0.18 | 0.38 | 0 | 1 | 1.69 | 0.85 | n.s. |
| **17** | 300 | 0.10 | 0.30 | 0 | 1 | 2.65 | 5.06 |  | 300 | 0.04 | 0.20 | 0 | 1 | 4.67 | 19.89 | 0.004 |
| **18** | 300 | 0.11 | 0.31 | 0 | 1 | 2.48 | 4.17 |  | 300 | 0.10 | 0.30 | 0 | 1 | 2.72 | 5.40 | n.s. |
| **19** | 300 | 0.36 | 0.48 | 0 | 1 | 0.58 | -1.67 |  | 300 | 0.31 | 0.46 | 0 | 1 | 0.83 | -1.31 | n.s. |
| **20** | 300 | 0.02 | 0.13 | 0 | 1 | 7.51 | 54.63 |  | 300 | 0.02 | 0.13 | 0 | 1 | 7.51 | 54.63 | n.s. |
| **21** | 300 | 0.07 | 0.26 | 0 | 1 | 3.35 | 9.28 |  | 300 | 0.06 | 0.23 | 0 | 1 | 3.82 | 12.60 | n.s. |
| **22** | 300 | 0.05 | 0.22 | 0 | 1 | 4.11 | 14.93 |  | 300 | 0.02 | 0.15 | 0 | 1 | 6.28 | 37.61 | n.s. |
| **23** | 300 | 0.02 | 0.15 | 0 | 1 | 6.28 | 37.61 |  | 300 | 0.02 | 0.14 | 0 | 1 | 6.82 | 44.7 | n.s. |
| **24** | 300 | 0.05 | 0.22 | 0 | 1 | 4.11 | 14.93 |  | 300 | 0.09 | 0.29 | 0 | 1 | 2.85 | 6.15 | n.s. |
| **25** | 300 | 0.03 | 0.18 | 0 | 1 | 5.17 | 24.85 |  | 300 | 0.06 | 0.23 | 0 | 1 | 3.82 | 12.6 | n.s. |
| **26** | 300 | 0.06 | 0.24 | 0 | 1 | 3.69 | 11.63 |  | 300 | 0.07 | 0.26 | 0 | 1 | 3.35 | 9.28 | n.s. |
| **27** | 300 | 0.08 | 0.27 | 0 | 1 | 3.17 | 8.05 |  | 300 | 0.03 | 0.18 | 0 | 1 | 5.17 | 24.85 | 0.020 |
| **28** | 300 | 0.16 | 0.37 | 0 | 1 | 1.85 | 1.41 |  | 300 | 0.12 | 0.33 | 0 | 1 | 2.28 | 3.21 | n.s. |
| **29** | 300 | 0.21 | 0.41 | 0 | 1 | 1.44 | 0.08 |  | 300 | 0.20 | 0.40 | 0 | 1 | 1.49 | 0.23 | n.s. |
| **30** | 300 | 0.18 | 0.38 | 0 | 1 | 1.69 | 0.85 |  | 300 | 0.19 | 0.39 | 0 | 1 | 1.57 | 0.47 | n.s. |
| **31** | 300 | 0.20 | 0.40 | 0 | 1 | 1.52 | 0.31 |  | 300 | 0.22 | 0.42 | 0 | 1 | 1.32 | -0.25 | n.s. |
| **32** | 300 | 0.13 | 0.33 | 0 | 1 | 2.23 | 3.00 |  | 300 | 0.07 | 0.25 | 0 | 1 | 3.46 | 9.98 | 0.013 |
| **33** | 300 | 0.17 | 0.38 | 0 | 1 | 1.72 | 0.95 |  | 300 | 0.14 | 0.35 | 0 | 1 | 2.03 | 2.11 | n.s. |
| **34** | 300 | 0.08 | 0.27 | 0 | 1 | 3.08 | 7.52 |  | 300 | 0.05 | 0.22 | 0 | 1 | 4.11 | 14.93 | n.s. |

Note: N = 300 (male) and 300 (female). The scores of Item 19 (reverse item) was reversed. Based on the results of Shapiro-Wilk tests and tests of homogeneity of variance, gender differences were investigated with Mann-Whitney U tests on Item 1, 2, 3, 7, 8, 9, 10, 12, 14, 16, 18, 19, 20, 21, 23, 26, 28, 29, 30, 31, and 33, and Brunner-Munzel tests on Item 4, 5, 6, 11,13,15,17,22,24,25,27,32, and 34.
