# Supplementary material for: Developing and validating the Japanese version of the Referential Thinking Scale: A cross-sectional study
Source: PLoS One. 2023 Jul 7;18(7):e0283416. doi: 10.1371/journal.pone.0283416 (PMC10328373; doi:10.1371/journal.pone.0283416)
Supplement: S3 Table — (DOCX) [file pone.0283416.s003.docx]

**S3 Table. The Japanese version of the Referential Thinking Scale (J-REF).**

日本語版Referential Thinking Scale (J-REF)

問題：次の文章を読み、ご自身に当てはまるかどうかを はい または いいえ のどちらかで回答してください。できる限り正直に、すべての項目に回答してください。なお、飲酒やドラッグ（マリファナ、LSD、コカイン等）による体験は含めず、ふだんのご自身に当てはまるかどうかを回答してください。

|  | items |
| --- | --- |
| 1 | 誰かが会話しているのを耳にすると、私の悪口を言っているのかもしれないと思うことがよくある。 |
| 2 | 道を歩いていると、人が私のことについて何か話していると思うことがよくある。 |
| 3 | 誰かが笑っているのを見ると、私のことを笑っているのかもしれないと思うことがよくある。 |
| 4 | 知らない人に笑われることが多い。 |
| 5 | 私が部屋に入ると、そこにいた人がそわそわしだすことが多い。 |
| 6 | 職場でふたりの人が話しているのを見ると、たいてい私のことを批判していると思う。 |
| 7 | ふたりの人が外国語で話しているのを聞くと、私のふるまいについて話しているのだと思うことがよくある。 |
| 8 | 知らない人に服装を見られていることが多い。 |
| 9 | 人に見られていると感じることがよくある。 |
| 10 | 私の好きな曲を聴くと、私のことを考えながら作られた曲だと思う。 |
| 11 | 私のことが書かれているように思える本を読んだことがある。 |
| 12 | 映画を見て私の人生とよく似ていると思うことがよくある。 |
| 13 | ラジオのDJは私だけのために選曲してくれているのではないかと思うことがよくある。 |
| 14 | 私が通り過ぎたときに人が私のことを笑っているかもしれないと思うことがよくある。 |
| 15 | 新聞記事には私だけに向けたメッセージがこめられていると時々思う。 |
| 16 | 私が急いで車を運転していると、たいてい信号は赤になる。 |
| 17 | 犬の近くにいると、その犬はしょっちゅう私に吠えるようだ。 |
| 18 | 電車やバスに乗っているとき、しばしばじっと見つめられているようだ。 |
| 19 | 道ゆく人が私を特別に意識しているとは思わない。（R） |
| 20 | 先生が私のためだけに授業しているように思えることがよくある。 |
| 21 | 口にこそ出さないが、他の人は私の頭がどれほどよいのだろうかといつも思っているようだ。 |
| 22 | 私が通り過ぎたとき、小動物が私に特別注目しているようだ。 |
| 23 | 知らない人に手を振られることが多い。 |
| 24 | 私の服装について他の人が噂していると思うことがよくある。 |
| 25 | 私の身なりはよく他の人に真似されるようだ。 |
| 26 | 私のしゃべり方は他の人に真似されると思うことがよくある。 |
| 27 | 高速に乗っているとき、私が下りる出口でなぜこれほど多くの人が下りるのだろうかと思うことがよくある。 |
| 28 | 私が恥ずかしがっていると、他の人には恥ずかしがる理由が分かっていることが多いと思う。 |
| 29 | 私が隠そうとしている性格は人にたいてい見破られる。 |
| 30 | 何か壊れているものを見ると、私のせいにされるかもしれないと思うことがよくある。 |
| 31 | 心の奥底では事実ではないと分かってはいても、何かあると私のせいにされていると感じることがよくある。 |
| 32 | なぜかは分からないが、私は非常に注目を浴びやすいようだ。 |
| 33 | 私のふるまいについてとがめられていると思うことがよくある。 |
| 34 | 私がこの集まりにいるから、他の人もこの集まりにいるのかもしれないと思うことがよくある。 |
|  | |

Note*.* (R): reverse item

Any alteration of instruction and items of J-REF are prohibited.

Translated from the original English version by Kotomi Kawaguchi and Jun Sasaki with permission from Mark F. Lenzenweger, PhD

Original English version: Copyright (c) 1996 by Mark F. Lenzenweger

Japanese version: Copyright (c) 2010 by Mark F. Lenzenweger
