# Supplementary material for: Developing and validating the Japanese version of the Referential Thinking Scale: A cross-sectional study
Source: PLoS One. 2023 Jul 7;18(7):e0283416. doi: 10.1371/journal.pone.0283416 (PMC10328373; doi:10.1371/journal.pone.0283416)
Supplement: S4 Table — (DOCX) [file pone.0283416.s004.docx]

|  |  |  |  |  |  |  |  |  |
| --- | --- | --- | --- | --- | --- | --- | --- | --- |
|  |  |  |  |  |  |  |  |  |
|  |  |  |  |  |  |  |  |  |
|  |  |  |  |  |  |  |  |  |
|  |  |  |  |  |  |  |  |  |
|  |  |  |  |  |  |  |  |  |
|  |  |  |  |  |  |  |  |  |
|  |  |  |  |  |  |  |  |  |
|  |  |  |  |  |  |  |  |  |
|  |  |  |  |  |  |  |  |  |
|  |  |  |  |  |  |  |  |  |
|  |  |  |  |  |  |  |  |  |
|  |  |  |  |  |  |  |  |  |

|  | **n** | **Means** | **SD** | **Min.** | **Max.** | **Skewness** | **Kurtosis** | **α** |
| --- | --- | --- | --- | --- | --- | --- | --- | --- |
| **J-REF** | 600 | 4.06 | 4.79 | 0 | 30 | 2.11 | 5.50 | .89 |
| **SRS** | 600 | 33.07 | 11.90 | 12 | 60 | 0.06 | -0.66 | .94 |
| **SC-PUB** | 600 | 48.79 | 11.93 | 11 | 77 | -0.37 | 0.46 | .90 |
| **SC-PRI** | 600 | 43.48 | 9.94 | 10 | 70 | -0.17 | 0.57 | .88 |
| **SPQ-CogPer** | 600 | 7.03 | 6.57 | 0 | 33 | 1.23 | 1.46 | .91 |
| **SPQ-Inter** | 600 | 12.98 | 8.42 | 0 | 33 | 0.25 | -0.91 | .93 |
| **SPQ-Disorg** | 600 | 5.01 | 4.17 | 0 | 16 | 0.68 | -0.40 | .88 |
| **SDS** | 600 | 47.24 | 9.41 | 21 | 77 | 0.09 | 0.07 | .85 |
| **STAI-S** | 600 | 47.06 | 11.54 | 20 | 80 | 0.14 | -0.03 | .92 |
| **NA** | 600 | 22.93 | 8.32 | 8 | 48 | 0.28 | 0.04 | .92 |
| **PA** | 600 | 21.32 | 7.01 | 8 | 43 | 0.11 | -0.11 | .88 |
| **SPS** | 600 | 18.32 | 17.56 | 0 | 80 | 1.21 | 0.99 | .96 |

**S4 Table. Descriptive statistics of the J-REF and other scales (n = 600).**

Note: N = 600. J-REF = Japanese version of Referential Thinking Scale; SC-PUB = Public Self-Consciousness Scale; SC-PRI = Private Self-Consciousness Scale; SRS = Self-Reference Scale; SPQ-CogPer = positive schizotypy; SPQ-Inter = negative schizotypy; SPQ-Disorg = disorganization; SDS = Self-rating Depression Scale; STAI-S = State-Trait Anxiety Inventory (A-State); NA = Negative Affect Scale; PA = Positive Affect Scale; SPS = Social Phobia Scale.
