# Supplementary material for: Developing and validating the Japanese version of the Referential Thinking Scale: A cross-sectional study
Source: PLoS One. 2023 Jul 7;18(7):e0283416. doi: 10.1371/journal.pone.0283416 (PMC10328373; doi:10.1371/journal.pone.0283416)
Supplement: S5 Table — (DOCX) [file pone.0283416.s005.docx]

|  |  |  |  |  |  |  |  |  |  |  |  |  |
| --- | --- | --- | --- | --- | --- | --- | --- | --- | --- | --- | --- | --- |
|  |  |  |  |  |  |  |  |  |  |  |  |  |
|  |  |  |  |  |  |  |  |  |  |  |  |  |
|  |  |  |  |  |  |  |  |  |  |  |  |  |
|  |  |  |  |  |  |  |  |  |  |  |  |  |
|  |  |  |  |  |  |  |  |  |  |  |  |  |
|  |  |  |  |  |  |  |  |  |  |  |  |  |
|  |  |  |  |  |  |  |  |  |  |  |  |  |
|  |  |  |  |  |  |  |  |  |  |  |  |  |
|  |  |  |  |  |  |  |  |  |  |  |  |  |
|  |  |  |  |  |  |  |  |  |  |  |  |  |
|  |  |  |  |  |  |  |  |  |  |  |  |  |
|  |  |  |  |  |  |  |  |  |  |  |  |  |
|  | **1** | **2** | **3** | **4** | **5** | **6** | **7** | **8** | **9** | **10** | **11** | **12** |
| **1 J-REF** |  | .56^***^ | .38^***^ | .26^***^ | .70^***^ | .51^***^ | .60^***^ | .43^***^ | .39^***^ | .45^***^ | -.12 | .51^***^ |
| **2 SRS** |  |  | .56^***^ | .27^***^ | .50^***^ | .48^***^ | .41^***^ | .45^***^ | .41^***^ | .48^***^ | -.21^***^ | .53^***^ |
| **3 SC-PUB** |  |  |  | .60^***^ | .36^***^ | .25^***^ | .28^***^ | .19^***^ | .21^***^ | .35^***^ | .02 | .33^***^ |
| **4 SC-PRI** |  |  |  |  | .31^***^ | .22^***^ | .25^***^ | .13^***^ | .14^*^ | .27^***^ | .08 | .23^***^ |
| **5 SPQ-CogPer** |  |  |  |  |  | .74^***^ | .77^***^ | .44^***^ | .42^***^ | .44^***^ | -.10^**^ | .55^***^ |
| **6 SPQ-Inter** |  |  |  |  |  |  | .76^***^ | .52^***^ | .51^***^ | .46^***^ | -.35^***^ | .54^***^ |
| **7 SPQ-Disorg** |  |  |  |  |  |  |  | .42^***^ | .42^***^ | .44^***^ | -.12^**^ | .48^***^ |
| **8 SDS** |  |  |  |  |  |  |  |  | .80^***^ | .59^***^ | -.45^***^ | .48^***^ |
| **9 STAI-S** |  |  |  |  |  |  |  |  |  | .63^***^ | -.41^***^ | .48^***^ |
| **10 NA** |  |  |  |  |  |  |  |  |  |  | -.03 | .47^***^ |
| **11 PA** |  |  |  |  |  |  |  |  |  |  |  | -.24^***^ |
| **12 SPS** |  |  |  |  |  |  |  |  |  |  |  |  |

**S5 Table. Correlation of the J-REF and other scales (n = 600).**

Note: N = 600. ^*^ *p* < .05, ^**^ *p* < .01, ^***^ *p* < .001. J-REF = Japanese version of Referential Thinking Scale; SC-PUB = Public Self-Consciousness Scale; SC-PRI = Private Self-Consciousness Scale; SRS = Self-Reference Scale; SPQ-CogPer = positive schizotypy; SPQ-Inter = negative schizotypy; SPQ-Disorg = disorganization; SDS = Self-rating Depression Scale; STAI-S = State-Trait Anxiety Inventory (A-State); NA = Negative Affect Scale; PA = Positive Affect Scale; SPS = Social Phobia Scale.
