# Supplementary material for: Developing and validating the Japanese version of the Referential Thinking Scale: A cross-sectional study
Source: PLoS One. 2023 Jul 7;18(7):e0283416. doi: 10.1371/journal.pone.0283416 (PMC10328373; doi:10.1371/journal.pone.0283416)
Supplement: S6 Table — (DOCX) [file pone.0283416.s006.docx]

|  | **J-REF(SRS)** | **SRS( J-REF)** |
| --- | --- | --- |
| **SC-PUB** | .10^*^ | .45^***^ |
| **SC-PRI** | .13^**^ | .16^***^ |
| **SPQ-CogPer** | .59^***^ | .19^***^ |
| **SPQ-Inter** | .33^***^ | .28^***^ |
| **SPQ-Disorg** | .48^***^ | .12^**^ |
| **SDS** | .25^***^ | .27^***^ |
| **STAI-S** | .22^***^ | .24^***^ |
| **NA** | .26^***^ | .30^***^ |
| **PA** | -.00 | -.17^***^ |
| **SPS** | .30^***^ | .34^***^ |

**S6 Table. Partial correlation of scales controlling for J-REF and SRS (n = 600).**

Note: N = 600. * p < .05, ** p < .01, *** p < .001. Control variable is in parenthesis. J-REF = Japanese version of Referential Thinking Scale; SC-PUB = Public Self-Consciousness Scale; SC-PRI = Private Self-Consciousness Scale; SRS = Self-Reference Scale; SPQ-CogPer = positive schizotypy; SPQ-Inter = negative schizotypy; SPQ-Disorg = disorganization; SDS = Self-rating Depression Scale; STAI-S = State-Trait Anxiety Inventory (A-State); NA = Negative Affect Scale; PA = Positive Affect Scale; SPS = Social Phobia Scale.
