# Supplementary material for: Developing and validating the Japanese version of the Referential Thinking Scale: A cross-sectional study
Source: PLoS One. 2023 Jul 7;18(7):e0283416. doi: 10.1371/journal.pone.0283416 (PMC10328373; doi:10.1371/journal.pone.0283416)
Supplement: Attachment — Submitted filename: Response_to_Reviewers.docx [file pone.0283416.s007.docx]

Reviewer #1:

#1-1

This is an interesting study, very well presented and developed. The authors are well aware of the fundamental aspects and importance of self-referential processing, particularly in the form of ideas of reference. Although it is in fact a psychometric study with indications of reliability and validity for the Japanese version of the REF referential thinking scale, a novel approach is also offered that differentiates ideas of reference related to paranoia (IoR-P), and related to the schizophrenia spectrum (IoR-S). It is considered a valuable work, and hopefully it will be the basis for longitudinal studies that will allow the deepening of the onset of delusional disorders, whether or not of referential type or referential content.
The introduction is well presented, highlighting the main aspects of the ideas of reference, although with a main focus on the contribution of Lenzenweger, after all, the main author in the development of the REF scale, indebted to Meehl's contributions. Although there are many contributions that could be pointed out on the ideas of reference, the approach of its authors seems to us coherent.
**Response**: Thank you so much for deeply understanding the significance of our manuscript. Your thoughtful comment and suggestion have really enriched our understanding on IoR and the possibility to differentiate between IoR-S and IoR-P. It is deeply appreciated. As you might know, many empirical studies on IoR have not necessarily been conducted so far, so the authors assume that my replies and revision, especially on the discussion, might inevitably have some kind of speculation. At the same time, the authors believe that this study will be the basis of further empirical studies on IoR and the difference between IoR-S and IoR-P.

#1-2

However, the social component of the IoR is perhaps mentioned in a very lateral way, being essential to its definition (external events and the action of others are alluded to). Moreover, the greater development or emphasis on the social characteristic would fit in with later analyses related to social anxiety. It is recommended that the text DOI:<https://journals.sagepub.com/doi/10.1177/2167702620951553> be analyzed, because of the importance of this social aspect, and it frames IoR not only in a pathological sense, but in the context of our development as a species.

**Response**: Thank you for the meaningful comments that enrich the importance of this manuscript. The authors have added the following paragraph to the discussion as this paragraph provides a deeper understanding of the results related to the social aspects (Line 628 to Line 634):

“The social aspects of the IoR are essentially critical. For example, Bell et al. [63] argued that the theme of delusions is mainly social (e.g., [64]), and similarly, delusion-like beliefs may arise from normal adaptive social processes (e.g. [66, 67]). In other words, having delusion-like beliefs is functionally rational [68], while the content is irrational. Hence, focusing on the social aspects of IoR is quite important because it broadens our perspective not only in the pathological aspects of this phenomenon, but also to its social adjustment benefits in healthy individuals.”

#1-3

In the introduction reference is made to a classic in the study of delusions, Ernst Kretschmer, and we share the importance of this historical mention. However, ideas of reference are emphasized in normal functioning and the adolescent stage, when it may be a more important reference with respect to the origin or basis of the reference sensitive delusion, which is admittedly rather transient (asthenic character), but also the possibility of evolution to paranoia (stenic character). Perhaps it would be better to specify this mention.
**Response**: Thank you for pointing this out. The authors realized that we mentioned IoR as “always transient and commonly occurs during the developmental stages of adolescence and young adulthood.” The authors also tried to mention that some transition to paranoia. We

revised the manuscript as follows (Line 42 to Line 45):

“A classic way of understanding IoR traces its origin to Ernst Kretschmer, who suggests that IoR is mainly transient and commonly occurs during the developmental stages of adolescence and young adulthood, although there are some shifts to paranoia. ”

#1-4
Also in the introduction, the more current approach of authors such as Sass, Parnas, Henriksen, Raballo... who point out the importance of alterations of the self, at the basis of schizophrenia, and therefore, would presumably be more linked to the IoR-S, is missing. In fact, these authors incorporate the ideas of reference in instruments such as the EASE for early or prodromal detection of schizophrenia. Perhaps a mention along these lines could strengthen the discussion, given this differentiation obtained in this study. <https://pubmed.ncbi.nlm.nih.gov/32105619/> may also be reviewed.
**Response**: Thank you for your meaningful comments, which helped improve our manuscript. The authors recognized that we missed the more recent contributions on the importance of alterations of the self. The authors have added some descriptions as follows (Line 97 to Line 100):

“More recently, self-disorders [18] especially contributes to the onset of Schizophrenia [19, 20]. For example, Examination of Anomalous Self-Experience scale covers IoR along with this kind of experiences [21]. In this meaning, IoR could indicate a potential risk of developing psychotic disorders. ”

#1-5

From the methodological and statistical analysis point of view, the work is very good, correct, flawless. The instrument preparation procedure, with all the steps followed, is excellent. Very careful steps have been taken for the preparation of the instrument items, an aspect of the work that should be highlighted.
**Response**: Thank you so much for approving our procedure of the statistical analyses.

#1-6

However, since they indicate a Heywood case, it is not clear how these participants are selected, was it therefore an ad-hoc decision, perhaps this should be clarified.
**Response**: Thank you for pointing this out. The online survey company that the authors asked to collect data, had to prepare for the possibility that the 2nd survey’s data was lacking, and thus made a subsample aside from the 300 sample. (The data not only had to meet all five inclusion criteria but also be collected twice for test-retest reliability). The authors used a sample provided from the company initially as requested (n=300) for EFA, but a Heywood case occurred. Then the authors randomly selected another 300 participants, and added from the subsample so that it would be the same ratio for both genders, thus avoiding gender bias. The authors have added this explanation about the sample as follows (Line 327 to Line 329):

“The online survey company collected additional data in the first survey as a subsample aside from the 300 participants, to ensure the confirmation of test-retest reliability using the sample that met the inclusion criteria.”

#1-7

It also generates doubt that both KMO procedures, it is assumed that, with the initial sample of 300 participants, and later of 600, values below the recommended values are obtained, is this correct? Or perhaps it is not sufficiently clear, in view of the degrees of freedom shown. This aspect, apart from the fact that the sample is limited, should be highlighted in the limitations of the work.
**Response**: Some researchers have experienced the ultra-low values for KMO calculated by tetrachoric correlations (please see below link). This study also uses the tetrachoric correlations for an EFA, because J-REF are responded by T-F scale. The authors think that this is the reason why the values are low, and yet they believe the KMO values of this study was suitable for factor analysis, based on Kaiser (1974). Therefore, the rationale of decision (i.e., Kaiser, 1974) and limitation related to KMO were added to the manuscript (Line 318 to Line 320; Line 341 to Line 344; Line 717 to Line 718, respectively).

<https://stats.stackexchange.com/questions/489957/huge-difference-in-kmo-statistic-based-on-type-of-correlation-pearson-vs-polych>

<https://www.researchgate.net/post/Why_is_my_KMO_changing>

“a Kaiser–Meyer–Olkin (KMO) value of 0.68 showed that our 34 items of the J-REF were suitable for factor analysis [49].”

“a KMO value of 0.62 showed that our 34 items of the J-REF were suitable for factor analysis [49]. A rule of thumb indicates that a lower value of KMO is calculated with tetrachoric correlations than Pearson’s correlations.”

“Fourth, the KMO values were suitable for factor analyses but were low. Replications of factor analysis is variable.”

#1-8

Authors are strongly recommended to review the format of the tables they include, following APA standards, apart from the fact that they are displayed altered, with information in the cells occupying more than one line, etc.
The discussion is good. Perhaps too much clinging to the results, with less comparison with what it means with respect to other works. For example, given that the results have many similarities to those obtained by Rodriguez-Testal et al, what are the implications given that it is not exactly what was achieved by Lenzenweger et al?

Perhaps, and tentatively, the aforementioned authors propose a relationship of IoR with aberrant salience (<https://www.frontiersin.org/articles/10.3389/fpsyg.2022.878331/full>). It would be interesting, based on the results obtained and the differentiation of IoR-P and IoR-S, what relationship do the authors suggest? It is clear that they are different methodologies and a different approach, but perhaps the authors can raise some idea for further studies. Is it possible that the stages described by Klaus Conrad particularly cover IoR-S?
**Response**: We apologize for the incorrect format. We believe that the revised manuscript is based on the correct format as the authors requested an English proofreading company to format the manuscript according to the PLOS ONE’s guidelines.

Regarding the relationship between our results and previous studies, we focused mainly on two representative studies using REF, Lenzenweger et al. (1997) and Rodríguez-Testal et al. (2019), and provided the information for the discussion as follows (Line 635 to Line 663).

“The results of this study share some similarities with previous studies using the REF scale. Consistent with Lenzenweger et al. [13], the present study suggests that positive schizotypy predicts IoR-S. More recently, Ceballos-Munuera, et al. [69] showed that aberrant salience has the mediating role between IoR and psychotic dimension. Aberrant salience [70] refers to the experience of normal events as remarkable, which is specific to schizotypy and Schizophrenia. The concept of aberrant salience might be a promising perspective to differentiate the etiology of IoR-S and IoR-P, and emphasize that some kind of self-disorder [18] could be involved in the manifestation of IoR-S. For example, Nelson et al. [71] assumed that aberrant salience contributed to hyper-reflexivity [72], which refers to “excessive attention being paid to aspects of experience that are normally tacit and remain in the ‘background’ of awareness” [71]. In differentiating the etiology of IoR-S and IoR-P, it might also be important to control aberrant salience as well as social anxiety.

Regarding the result of CFAs, this study sheds light on the nature of IoR. Similar to Rodriguez-Testal et al. [27], this study supported a model of one second-order factor and five first-order factors using the oblique rotation method and indicated a better fit than the unidimensional model, although both models’ fit indices were high enough but slightly different. Contrarily, Lenzenweger et al. [13] showed the first-order factor model using varimax rotation. Thus, this suggests that the subscales of REF highly correlate with each other and that each subscale of REF could represent subtle but meaningful qualitative differences. Indeed, Lenzenweger et al. intended to cover relevant experiences, such as intrapersonal and interpersonal reflection, toward persons or animals with relatively positive, neutral, or negative valences, and so on [13]. However, a true-false scale that the original and Japanese version of REF employed have difficulties as an ordinal scale for the statistical analysis, such as factor analysis and reliability, as shown in this study. As mentioned previously, responding to the SPQ on a five-point Likert scale makes the reliability higher than a true-false scale [58]. Hence, further exploring the proper response style would result in understanding the nature of IoR better. ”

As you suggested, the authors have also considered the aberrant salience would contribute to the manifestation of IoR-S, and be a key concept that differentiates IoR-S and IoR-P. In the manuscript, the authors pointed out the importance of aberrant salience on the manifestation of IoR-S and added some explanations as follows (Line 635 to Line 647).

“The results of this study share some similarities with previous studies using the REF scale. Consistent with Lenzenweger et al. [13], the present study suggests that positive schizotypy predicts IoR-S. More recently, Ceballos-Munuera, et al. [69] showed that aberrant salience has the mediating role between IoR and psychotic dimension. Aberrant salience [70] refers to the experience of normal events as remarkable, which is specific to schizotypy and Schizophrenia. The concept of aberrant salience might be a promising perspective to differentiate the etiology of IoR-S and IoR-P, and emphasize that some kind of self-disorder [18] could be involved in the manifestation of IoR-S. For example, Nelson et al. [71] assumed that aberrant salience contributed to hyper-reflexivity [72], which refers to “excessive attention being paid to aspects of experience that are normally tacit and remain in the ‘background’ of awareness” [71]. In differentiating the etiology of IoR-S and IoR-P, it might also be important to control aberrant salience as well as social anxiety. ”

#1-9
The authors are congratulated for an interesting, novel and relevant article and, as noted in the previous paragraph, perhaps it is important to propose dynamic aspects so that this differentiation between IoR-S and IoR-P does not remain exclusively a separation of statistical adjustment in regression procedures.

**Response**: Thank you for your suggestion on the dynamic aspects on IoR-S and IoR-P from this study’s results and discussion. We added this as follow (Line 674 to Line 686).

“Considering the findings of this study, the dynamic manifestation process of IoR-S and IoR-P could be delineated: Individuals can experience IoR-S and IoR-P by enhancing social anxiety when one encounters a social situation. The aspect towards which one’s attention shifts, would determine the extent of IoR-P, not IoR-S—If it is their external aspects (i.e., public self-consciousness) or their internal state (i.e., private self-consciousness), IoR-P would be increased or decreased, respectively. However, IoR-S would not be influenced by the public and private self-consciousness. Those with more experience that normal people usually do not experience (i.e., positive schizotypy) and who cannot organize their thoughts and actions (i.e., disorganization) would manifest more IoR-S. As stated previously, schizotypy is formed from the interaction of genetic factors and social learning schedules [26]. A longitudinal study is needed to clarify what kind of social events could contribute to the manifestation of IoR-S and IoR-P, when encountered by individuals with the genetic background of schizotypy.”

Reviewer #2:

#2-1

The authors present a very interesting paper in which they describe the reliability and validity evidence for the Japanese version of the Referential Thinking Scale (REF) and present some analyses of variables that predict ideas of reference. Overall the paper is well written, but I am concerned about some methodological and statistical issues. Here are some comments that, in my opinion, could be used to improve the paper.

Introduction

The introduction addresses the constructs of interest and describes the association between referential thinking with psychotic and schizotypal symptoms among others. Findings found by other authors who have employed the REF scale of referential thinking are mentioned and the objectives and hypotheses are stated.

I found the presentation of the objectives and hypotheses unclear and disorganized.

The psychometric objectives are not clearly specified; it is not sufficient to state that reliability and validity are to be examined. This should be described with greater precision.

In the hypotheses of the second objective, too many justifications are included as to why the hypotheses are based on the results of other researchers; this generates confusion in the reading. These hypotheses should be described more clearly.
**Response**: Thank you for pointing out the description about the aim and hypothesis. As for the first aim (examining reliability and validity), the authors have tried to clearly mention “convergent” or “discriminant” validity in the purpose section and make the hypothesis clearer. Furthermore, the authors have realized that the expression and citations in the introduction could cause misunderstandings. When we mentioned in old version of manuscript (Line 151 to Line 152) “as prior research has shown” in hypothesizing the correlation of J-REF with other scales, we intended to mention “as prior research that developed and validated the REF,” because we believe it is natural to assume the same correlation patterns with the previous validation study. We have added these points as follows (Line 145 to Line 152):

“To investigate these questions, the present cross-sectional study aimed to develop the Japanese version of the REF (J-REF) and examine its convergent validity, discriminant validity, and reliability. Based on prior research that developed and validated the REF [13, 26], we hypothesized as follows:

(1) J-REF would be more strongly and positively correlated with IoR and positive schizotypy than with negative schizotypy and disorganization.
(2) J-REF would have a weaker association with self-consciousness, mood, depression, and anxiety than with schizotypy dimensions. ”

Regarding the second aim, our study is basically an exploratory study on the predictor of IoR-S and IoR-P; we did not have a strong hypothesis and tried to investigate them by controlling the confounding factor (i.e., social anxiety). To express this, the authors used the term “to explore” in the old manuscript and explained why social anxiety is important in the second aim. However, your comment helped us to understand the proper arrangements and appropriate emphasis on our claim. Therefore, we have changed some expressions as follows (Line 156 to Line 173):

“The second aim was to investigate the predictors of the manifestation of IoR-P and IoR-S, exploratorily. As mentioned previously, there are mixed results in the literature regarding the prediction of IoR manifestation [e.g., 10, 13, 27]. This might have been caused by a covariate factor that was uncontrolled in analyzing the relationship between IoR and other constructs. In fact, Brown et al. [31] highlighted the methodological difficulty in addressing the relationship between psychopathologies that share some points with each other. In this study, the Social Phobia Scale (SPS) [32] was used as one of covariates, considering that the nature of IoR-P and IoR-S share the feature of misunderstanding that one is being observed by others. As previously stated, Stefanis et al. [4] demonstrated a relationship between IoR-P and social anxiety. Morrison and Cohen [33] mentioned that SPS is more appropriate in assessing the fear of being observed by others, while the Social Interaction Anxiety Scale [32] represents the fear of negative evaluation in the situation of being observed by others—Would public self-consciousness solely predict the manifestation of IoR-P? Alternatively, would positive schizotypy solely predict the manifestation of IoR-S? Furthermore, if the interaction effects were significant, public self-consciousness or positive schizotypy would moderate the manifestation of IoR. Hence, these findings would enrich our knowledge of the etiology of IoR. ”

#2-2

Method
The way in which the REF scale was disseminated is not described, was it through social networks, posters, bulletin boards, etc. ....? What type of population responded to the questionnaire, were they university students, people from the general population?

How was it controlled that 150 males and 150 females responded? Were there more than 300 responses to the survey and were subjects eliminated for gender balance?

**Response**: Thank you for pointing this out. The authors wrote the process of recruit and inclusion criteria in old manuscript as well (Line 174 to Line 197). Now we wrote this point in the “Participants and procedure” section that an online survey company collected data that met the five inclusion criteria, including criteria (c) “gender such that an equal number of males and females was obtained”. This means the online survey company randomly selected and provided the data with 150 males and 150 females to us in order to eliminate gender bias. (Line 177 to Line 189):

“A total of 300 participants (150 females and 150 males) aged 20–29 years (Mage = 25.57 years, SD = 2.70) were selected through an online survey company (Cross-Marketing, Inc.). Tinsley and Tinsley [34] recommended a ratio of five to ten participants per item for factor analysis. As the REF has 34 items, 170–340 participants would suffice. In addition to budgetary restrictions, the sample size for the survey was determined. After four weeks, a random subgroup of 150 participants (75 females and 75 males; Mage = 25.41 years, SD = 2.78) responded to the J-REF again to assess test-retest reliability. All participants were recruited during March to April 2022.

The inclusion criteria were as follows: (a) residence in Japan; (b) people in their 20’s, who were of similar age to individuals enrolled in the study by Lenzenweger et al. [13]; (c) gender such that an equal number of males and females was obtained; (d) those who correctly responded to an instructional manipulation check [35]; and (e) those who correctly responded to an age consistency check. ”

#2-3
In the "measures" section put the full title of the scale with the respective citation, not the acronyms, even if you have previously specified the meaning of the acronyms.
**Response**: Thank you for the suggestion. The authors have corrected this by referring to the guideline and previous articles in Plos One.

#2-4
In the description of the REF scale, provide data on the psychometric properties of the instrument as found by the authors of the scale.
**Response**: Thank you for the meaningful suggestion regarding the psychometric properties of the original REF. The authors added the following points to the manuscript, although Lenzenweger et al. (1997) did not report the alpha coefficients of each subscale as follows (Line 217 to Line 221):

“The original version of REF has high internal reliability (α = .85) and high four-week test-retest reliability (r = .86) for the total REF score. It also indicates high construct validity, which shows a stronger correlation of REF with the schizotypal constructs, such as perceptual aberration (r = .53) and magical ideation (r = .61), and no significant correlation with self-consciousness (r = -.10) [13].”

#2-5

Results
Before performing the factor analysis, it would have been interesting if the authors had presented descriptive analyses of the responses to the REF scale items, as well as a contrast of means by sex.
**Response**: Thank you for commenting about the descriptive statistics regarding the items of J-REF. The result of EFA (“Model 3” in Table of CFA) was obtained by 600 participants; therefore, the authors have newly added two supplemental tables using 600 participants (S1 Table: descriptive statistics and S2 Table: each gender) as supplementary information before the section of conducting EFA. Considering this change, the serial number of the supplemental tables have been changed from the previous manuscript.

#2-6

I do not understand the authors' description from line 320 to line 329. Was the initial sample composed of 600 participants and they selected 300? If this is so, why was it not described in the methodology section. If 300 participants were randomly selected, they must show that this selection does not present significant differences in sociodemographic and clinical variables with respect to the unselected sample.

Table 1 states that the n is 600 participants, wasn't it 300? Again, this shows that the description of the sample is not at all clear.
**Response**: Thank you for pointing this out. The online survey company that the authors asked to collect data, had to prepare for the possibility that the 2nd survey’s data was lacking, and thus made a subsample aside from the 300 sample. (The data not only had to meet all five inclusion criteria but also be collected twice for test-retest reliability). The authors used a sample provided from the company initially as requested (n=300) for EFA, but a Heywood case occurred. Then the authors randomly selected another 300 participants, and added from the subsample so that it would be the same ratio for both genders, thus avoiding gender bias. The authors have added this explanation about the sample as follows (Line 327 to Line 329):

“The online survey company collected additional data in the first survey as a subsample aside from the 300 participants, to ensure the confirmation of test-retest reliability using the sample that met the inclusion criteria.”

#2-7

To perform the validation of an instrument they should randomly select half of the sample and perform an AFE with one half and a CFA with the other half of the sample. Cross-validation.
**Response**: I understand “AFE” as EFA. As the authors mentioned in manuscript, a Heywood case can occur from the lack of sample in EFA, and selecting half of sample for EFA would heighten the risk of a Heywood case. Thank you for your suggestion.

#2-8

The descriptive results of the scales presented in Table 3 should be presented in an annex, except for those of the REF scale, which can be described in the text.
**Response**: The authors think that it is important to show the descriptive feature of scales other than J-REF, because they appear later in the analyses and show the different tendencies in comparative results, such as hierarchical regression analyses or partial correlations. It would also help the readers to understand the relevant constructs that we addressed in this study. Hence, the authors did not change the information in Table 3. Thank you for your suggestion.

#2-9

In the hierarchical regression analyses present in addition to VIF, tolerance.
Even if the VIF results do not indicate multicollinearity, why include the reference ideas in the first step of the analyses, what is the point?
**Response**: Thank you for pointing this out. The authors have added this point in the manuscript along with the reference criteria of the statistical value as follows (Line 483 to Line 484):

“The VIF ranged from 1.34 to 3.94, and tolerance, which is the reciprocal of VIF ranged from 0.25 to 0.74, thus showed no multicollinearity issues [53]. ”

The reason for the reference ideas in the first step is that J-REF and SRS has strong correlation (r=0.6), which means, they share some features. This made it impossible to understand the specific moderator for IoR-P and IoR-S, as the authors mentioned in Line 489 to Line 492.

“Furthermore, there was a strong correlation between the J-REF and SRS (r = 0.60, p < .001). This shows the overlapping nature of IoR-S and IoR-P. To reveal the specific features of IoR-S, the SRS score was included in step 1 as a covariate, and the change in R2 was 0.36.”

#2-10

Discussion
The first sentence describing the objectives of the study should be more precise, as it responds vaguely to the results that have been carried out.
**Response**: Thank you for pointing out the need to clarify the objective of the study. We have revised the manuscript as follows (Line 526 to Line 529):

“The purpose of the present study was two-fold. The first purpose was to develop the J-REF and examine its convergent validity, discriminant validity, and reliability. The second purpose was to investigate the predictors of the manifestation of IoR-P and IoR-S by controlling covariates, exploratorily. ”

#2-11
This section adequately addresses what is proper for a discussion. It contrasts the results achieved with those found by other authors, presents the limitations and future lines of action and describes the contribution of the results of the work to the literature on the subject.

**Response**: Thank you for your comment. We added the difference from previous studies (Line 635 to Line 663) as follows:

“The results of this study share some similarities with previous studies using the REF scale. Consistent with Lenzenweger et al. [13], the present study suggests that positive schizotypy predicts IoR-S. More recently, Ceballos-Munuera, et al. [69] showed that aberrant salience has the mediating role between IoR and psychotic dimension. Aberrant salience [70] refers to the experience of normal events as remarkable, which is specific to schizotypy and Schizophrenia. The concept of aberrant salience might be a promising perspective to differentiate the etiology of IoR-S and IoR-P, and emphasize that some kind of self-disorder [18] could be involved in the manifestation of IoR-S. For example, Nelson et al. [71] assumed that aberrant salience contributed to hyper-reflexivity [72], which refers to “excessive attention being paid to aspects of experience that are normally tacit and remain in the ‘background’ of awareness” [71]. In differentiating the etiology of IoR-S and IoR-P, it might also be important to control aberrant salience as well as social anxiety.

Regarding the result of CFAs, this study sheds light on the nature of IoR. Similar to Rodriguez-Testal et al. [27], this study supported a model of one second-order factor and five first-order factors using the oblique rotation method and indicated a better fit than the unidimensional model, although both models’ fit indices were high enough but slightly different. Contrarily, Lenzenweger et al. [13] showed the first-order factor model using varimax rotation. Thus, this suggests that the subscales of REF highly correlate with each other and that each subscale of REF could represent subtle but meaningful qualitative differences. Indeed, Lenzenweger et al. intended to cover relevant experiences, such as intrapersonal and interpersonal reflection, toward persons or animals with relatively positive, neutral, or negative valences, and so on [13]. However, a true-false scale that the original and Japanese version of REF employed have difficulties as an ordinal scale for the statistical analysis, such as factor analysis and reliability, as shown in this study. As mentioned previously, responding to the SPQ on a five-point Likert scale makes the reliability higher than a true-false scale [58]. Hence, further exploring the proper response style would result in understanding the nature of IoR better. ”

The limitations are as follows (Line 706 to Line 721):

“This study had some limitations. First, it explored the specific predictors of two distinct types of IoR; however, it was a cross-sectional study. Thus, a longitudinal study is required. Second, the results of this study were established in a community sample, and it is not clear whether the findings of this study can be applied to patients with IoR, although it is known that people with psychiatric diagnoses have more IoR. Furthermore, the mechanisms of manifestation of IoR in various groups, such as those with particularly high schizotypy traits (i.e., high schizotypy [74]) and those with high public self-consciousness only and low schizotypy, are also unclear. It is necessary to investigate how the predictors and confounders identified in the present study engage in the manifestation of IoR. Third, the study was limited to a community sample of people in their 20s. A systematic study with various age groups is needed to determine the predictors of IoR-P and IoR-S in other age groups. Fourth, the KMO values were suitable for factor analyses but were low. Replications of factor analysis is variable. Finally, we were unable to confirm the internal consistency in one of the five subscales ("Songs, Newspapers, Books“). If the subscales are to be used in the analysis, it is necessary to confirm whether similar factors can be replicated in a Japanese sample.”

Thank you for considering our manuscript with PLOS ONE.
